# Transcriptional and Functional Insights Into the All‐Stage Resistance Gene *GmRmd1*‐Mediated Regulation of Resistance to Powdery Mildew in Soybean

**DOI:** 10.1111/mpp.70330

**Published:** 2026-08-28

**Authors:** Guoqiang Liu, Chen Ling, Tingting Xu, Xuehui Zhang, Jiacan Jiang, Xiaomin Xu, Lu Li, Jiajian Zhang, Bing Hu, Mengshi Liu, Yunxin He, Cunyi Yang

**Affiliations:** ^1^ Guangdong Provincial Key Laboratory of Plant Molecular Breeding, College of Agriculture South China Agricultural University Guangzhou China; ^2^ Yuelushan Laboratory/Institute of Cotton and Sericulture Hunan Academy of Agricultural Sciences Changsha China; ^3^ Key Laboratory for Enhancing Resource Use Efficiency of Crops in South China, Ministry of Agriculture and Rural Affairs South China Agricultural University Guangzhou China; ^4^ South China Institute for Soybean Innovation Research South China Agricultural University Guangzhou China

**Keywords:** gene mapping, GmRmd1, lignin, PMD, protein interaction, RNA‐Seq

## Abstract

Powdery mildew disease (PMD), caused by *Microsphaera diffusa*, is a devastating soybean foliar disease. This study identified *GmRmd1* as a candidate in Guangxi landrace MSRF (all‐stage resistance to PMD) and validated it as a positive PMD resistance regulator. Hypersensitive response (HR) assays confirmed its Toll interleukin‐1 receptor (TIR), nucleotide‐binding site (NBS) and basic secretory protein (BSP) domains are critical for immune responses. *GmRmd1* was induced by *M. diffusa* , and its expression was inhibited by *GmWRKY27* and *GmERF138* specifically binding to the W‐box and GCC‐box elements of the *GmRmd1* promoter, respectively. RNA‐Seq revealed that chromosome 16 (chr16) resistance cluster genes (*Glyma.16G214100*, *Glyma.16G214200*, *Glyma.16G215100* and *Glyma.16G215800*) showed *GmRmd1*‐related expression patterns. Yeast two‐hybrid, bimolecular fluorescence complementation and luciferase assays demonstrated GmRmd1 interacts not only with itself, but also with GmRmd2 (Glyma.16G215100), GmRmd3 (Glyma.16G214900) and GmRnl1 (NLR cofactor). In addition, mutants of *GmRmd3* and *GmRnl1* are more susceptible to *M. diffusa* . Notably, GmRmd1 enhances the resistance of PMD by interacting with GmPER1 (lignin synthesis‐related) and increasing the synthesis of lignin. Genomic analysis indicated that the number of soybean accessions carrying the *GmRmd1* locus gradually decreases from north to south and from west to east in China. Taken together, GmRmd1 forms functional protein complexes with GmRmd2, GmRmd3, and GmRnl1 to regulate lignin biosynthesis and thereby modulate disease resistance. These insights provide novel perspectives into the molecular mechanisms of *GmRmd1*‐mediated resistance to soybean PMD.

## Graphical abstract

*GmRmd1* is a soybean all‐stage powdery mildew (PMD) resistance gene. Under normal conditions, *GmWRKY27* and *GmERF138* suppress the expression of *GmRmd1*. Upon *Microsphaera diffusa* infection, GmRmd1 interacts with GmRmd2, GmRmd3, GmRnl1 and GmPER1, forming functional protein complexes to regulate lignin biosynthesis and thereby modulate disease resistance.

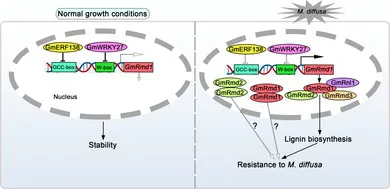

## 1 Introduction

Soybean ( *Glycine max* ) as a critical crop serves as the primary source of edible oil and plant protein for human consumption and livestock (Tyug et al. 2010; Sun et al. 2019). Powdery mildew disease (PMD), caused by the biotrophic airborne fungus *Microsphaera diffusa*, is a regional and seasonal disease under conditions of cool temperatures, high humidity and significant diurnal temperature variations. PMD manifests as white, powdery patches on seeds, stems, leaves and roots, and can lead to defoliation, venous chlorosis, necrosis or a combination thereof (Leath and Carroll 1982). This can result in yield losses of up to 40% in susceptible soybean cultivars when exposed to pathogens (Gonçalves et al. 2002).

The use of resistant cultivars represents the most effective and economically viable strategy for controlling PMD. Genetic resistance can be categorised into all‐stage resistance (ASR) and adult plant resistance (APR), according to the plant growth stage when resistance is phenotypically expressed. ASR is effective across all growth stages from seedling emergence, while APR typically provides intermediate levels of resistance that are more pronounced in the phenotypic expression at adult growth stages (Zadoks 1974).

Since the first disease‐resistant locus for PMD in soybean was identified in 1993 (Lohnes et al. 1993), all identified PMD‐resistance genes have been mapped to the distal end of soybean chr16. Resistance to PMD in soybean is primarily governed by three alleles located at the *Rmd* locus: *Rmd*‐c, *Rmd* and *rmd*. The genes *Rmd*‐c and *Rmd* act as dominant alleles for *rmd*. Materials carrying *Rmd‐c* demonstrate resistance to PMD throughout the soybean growth stage and exhibit complete resistance under field conditions; plants carrying *Rmd* are susceptible during the seedling stage but develop resistance post‐adult stage and show field resistance; only materials carrying *rmd* remain susceptible (Mignucci and Lim 1980; Sullivan 2003). The disease resistance locus *Rmd* was mapped using RFLP markers to the J linkage group at the end of chr16 and is closely linked to the *Rps2* locus, which confers resistance to soybean root rot (Polzin et al. 1994). The resistance locus in the PMD‐resistant accession PI243540 is localised between the SSR marker Sat224 and the SNP marker BARC‐021875‐04228 at the end of chr16 in the soybean genome, based on SSR and SNP marker analyses. The locus exhibits genetic distances of 9.6 and 1.3 cM from Sat224 and BARC‐021875‐04228, respectively (Kang and Mian 2010). A recombinant inbred line (RIL) population of the F_7_ generation was developed, consisting of 334 inbred lines, using the PMD‐susceptible accession Wyandot and the resistant accession PI567301B. The PMD resistance locus has been localised to a 1.4 cM interval flanked by the markers BARCSOYSSR_16_1272 and BARCSOYSSR_16_1298 (Jun et al. 2012). In a separate study, 53 F_2:3_ lines derived from a cross between the resistant accession V97‐3000 and the susceptible accession V99‐5089 were used to map the PMD resistance locus from V97‐3000 to a chromosomal region between markers Satt547 and Sat_396, located at the end of chr16, with genetic distances of 3.8 and 3.9 cM, respectively (Wang et al. 2013). Three mapping populations were developed by crossing resistant accession B3, Fudou234 and B13 with a susceptible accession to identify the location of the resistance locus. The resistance locus in B3 was localised between the SSR markers GMES6959 and Satt_393 at the end of chr16, with genetic distances of 7.1 and 4.6 cM, respectively. Subsequently, a high‐density genetic map was constructed using 248 F_8_ RILs derived from Guizao1 × B13, enabling the precise localisation of the PMD resistance locus in B13 to an 188.06 kb genomic region on chr16. This region encompasses 28 genes, among which 17 are potential candidate resistance (R)‐like genes (Jiang et al. 2019). A genome‐wide association study (GWAS) was performed using data from 315 soybean accessions to investigate resistance to PMD. Several significantly associated loci were mapped to a 156 kb region located at the end of chr16, and the region harbours a cluster of 19 R‐like genes potentially associated with PMD resistance (Liu, Fang, et al. 2024). Through fine mapping on the high‐density genetic linkage map of the F_1_, F_2_ and F_8:11_ RIL populations derived from the cross between Zhonghuang 24 (ZH24) and Huaxia 3 (HX3), the resistance locus responsible for the APR in ZH24 was narrowed down to a 32.8 kb genomic region at the end of chr16, encompassing five candidate genes (Zhou et al. 2022). Additionally, GWAS was conducted using a panel of 467 soybean accessions, in conjunction with fine mapping of 471 F_8_ RILs generated from the cross between the ASR accession B13 and Guizao 1, leading to the identification and functional validation of the first PMD resistance gene, *GmRmd1* (Xian et al. 2022). A further GWAS of 315 soybean cultivars identified a gene cluster on chr16 comprising 21 candidate genes associated with PMD resistance (Liu, Yang, et al. 2024). Haplotype analysis further revealed single‐nucleotide polymorphisms (SNPs) and insertion–deletions (InDels) within the promoters and coding regions of *Glyma.16G214000*, *Glyma.16G214200*, *Glyma.16G214300*, *Glyma.16G215100* and *Glyma.16G215300* that are significantly correlated with PMD resistance (Liu, Yang, et al. 2024).

Although the PMD resistance gene of the ASR accession B13 has been identified as *GmRmd1* (Xian et al. 2022), it is still uncertain whether *GmRmd1* is also responsible for PMD resistance in other resistant accessions, and the underlying mechanism by which *GmRmd1* confers resistance to PMD remains to be elucidated. In this study, a PMD resistance gene in soybean was mapped to *GmRmd1* using the ASR accession MSRF and the susceptible accession HX3 as parental materials. Additionally, the regulatory mechanism of *GmRmd1* in soybean resistance to PMD was explored through functional verification, transcriptome analysis and molecular interaction assays, providing robust theoretical support for the molecular design of disease‐resistant accession resources.

## 2 Results

### 2.1 Fine Mapping of MSRF Resistance Gene

To identify and map resistance genes of MSRF, we developed a mapping population named MH by crossing MSRF (resistant) as the maternal parent with HX3 (susceptible) as the paternal parent (Figure S1). The F_1_ offspring were resistant to *M. diffusa* . In the F_2_ generation, 1709 out of 2284 were resistant and 575 were susceptible to PMD, which fits an expected 3:1 segregation ratio (*χ* ^2^ = 0.04; *p* = 0.85) (Table S1). Moreover, among 194 F_2:3_ lines, 51 were homozygous resistant, 52 were homozygous susceptible and 91 were segregating for resistance, which fits an expected 1:2:1 segregation ratio (*χ* ^2^ = 2.15; *p* = 0.34) (Table S1). These results indicate that the resistance to *M. diffusa* in MSRF is conferred by a single dominant gene, which we tentatively named *Rmd_MSRF*.

**FIGURE 1:**
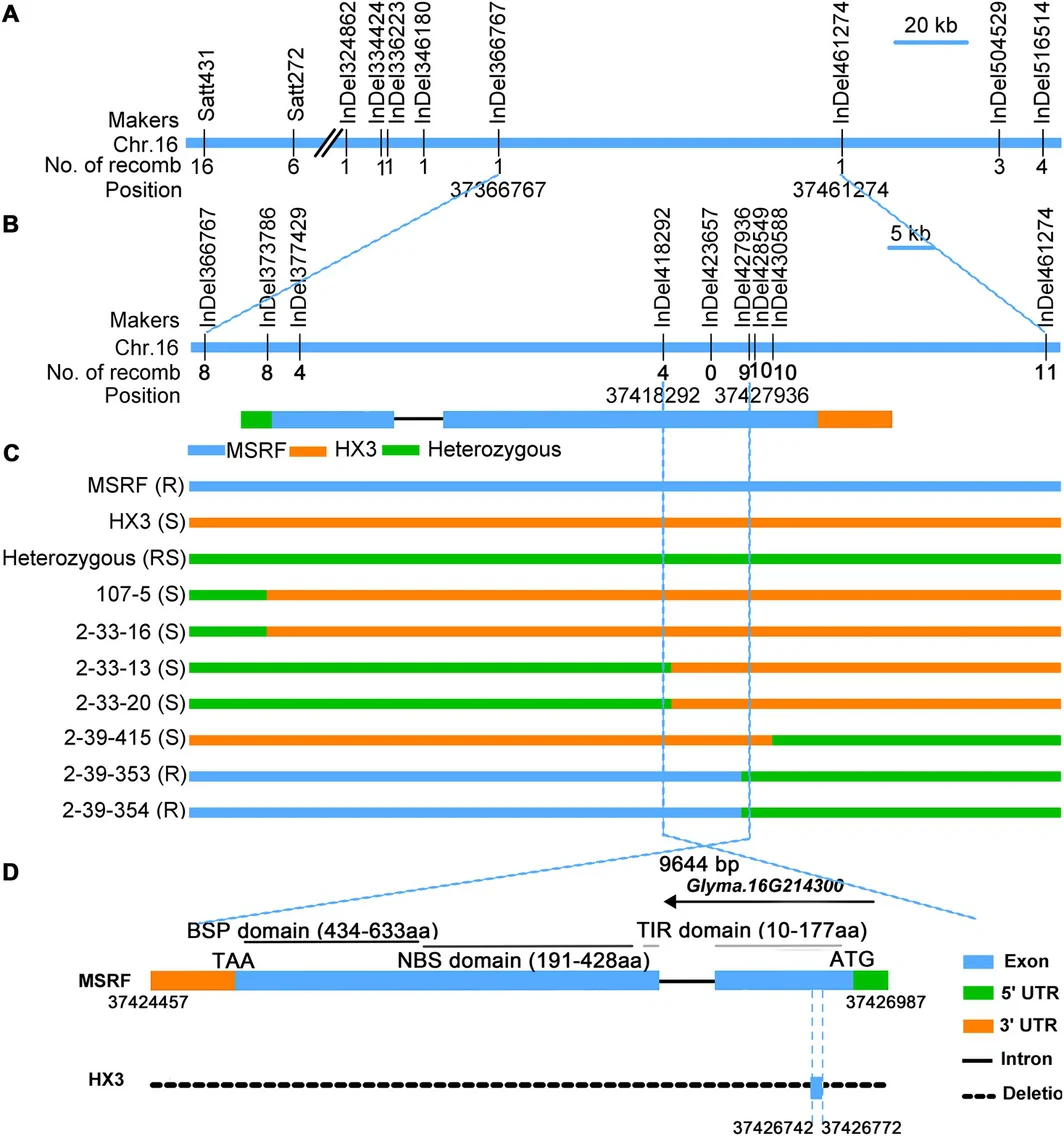
Mapping of the powdery mildew disease resistance gene in the MH population. (A) The preliminary mapping of the MH. (B) The fine mapping of the MH. (C) Recombinant plants of the fine mapping of the MH, R: Resistant; S: Susceptible. (D) Sequence analysis of *GmRmd1* in MSRF and HX3.

Previous GWAS analyses conducted in our laboratory revealed that the majority of resistance genes were clustered at the distal end of chr16 (Liu, Fang, et al. 2024). Consequently, simple‐sequence repeat (SSR) and InDel primers, with *G. max* Wm82.a4.v1 as the reference genome, were designed in proximity to the peak of the GWAS signal. This approach aimed to preliminarily map the location of resistance gene(s) within the MH population. We initially used primer pairs targeting Satt431, Satt272, InDel373786, InDel418292, InDel504529 and InDel516514 (Table S2) to amplify DNA from the 194 F_2:3_ lines. Combined with the phenotypic data, linkage analysis was carried out and the results showed that *Rmd_MSRF* was located between the Satt431 and InDel516514 markers (Figure S2 and Table S3). We then used 12 primer pairs targeting (Table S2) to amplify DNA from 10 informative recombinant lines (Table S4). This fine mapping placed *Rmd_MSRF* in a 94.4 kb interval between InDel366767 and InDel461274 (Figure 1A).

In order to identify a single PMD resistance gene, lines 107, 2–33 and 2–39, which are heterozygous for the InDel366767 and InDel461274 markers, were used to produce F_4_, F_5_ and F_6_ segregating populations. Six pairs of primers were used to map the recombinant plants, and *Rmd_MSRF* was mapped to a 9590 bp interval between InDel418292 and InDel427936 (Figure 1B,C and Table S4). Visualisation of this region using the Integrative Genomics Viewer (IGV) indicated that, in comparison to MSRF, HX3 exhibited extensive sequence loss within this segment (Figure S3). This interval contains only one gene (*Glyma.16g214300*) named *GmRmd1* (Figure 1A–D). In another study, it was also discovered that the resistance gene in the resistant accession B13 is *GmRmd1* (Xian et al. 2022) and when compared to B13, the *GmRmd1* in MSRF differs by only a few amino acids (Figure S4). *GmRmd1* has homology to the TNX gene family, which encodes plant‐specific proteins that recognise pathogen effectors. In GmRmd1, the conserved LRR domain is replaced by a BSP domain, which together with the canonical TIR and NBS domains creates a novel combination of domains in the TNX family (Figure 1D).

### 2.2 *GmRmd1* Positively Regulates PMD Resistance in Soybean

**FIGURE 2:**
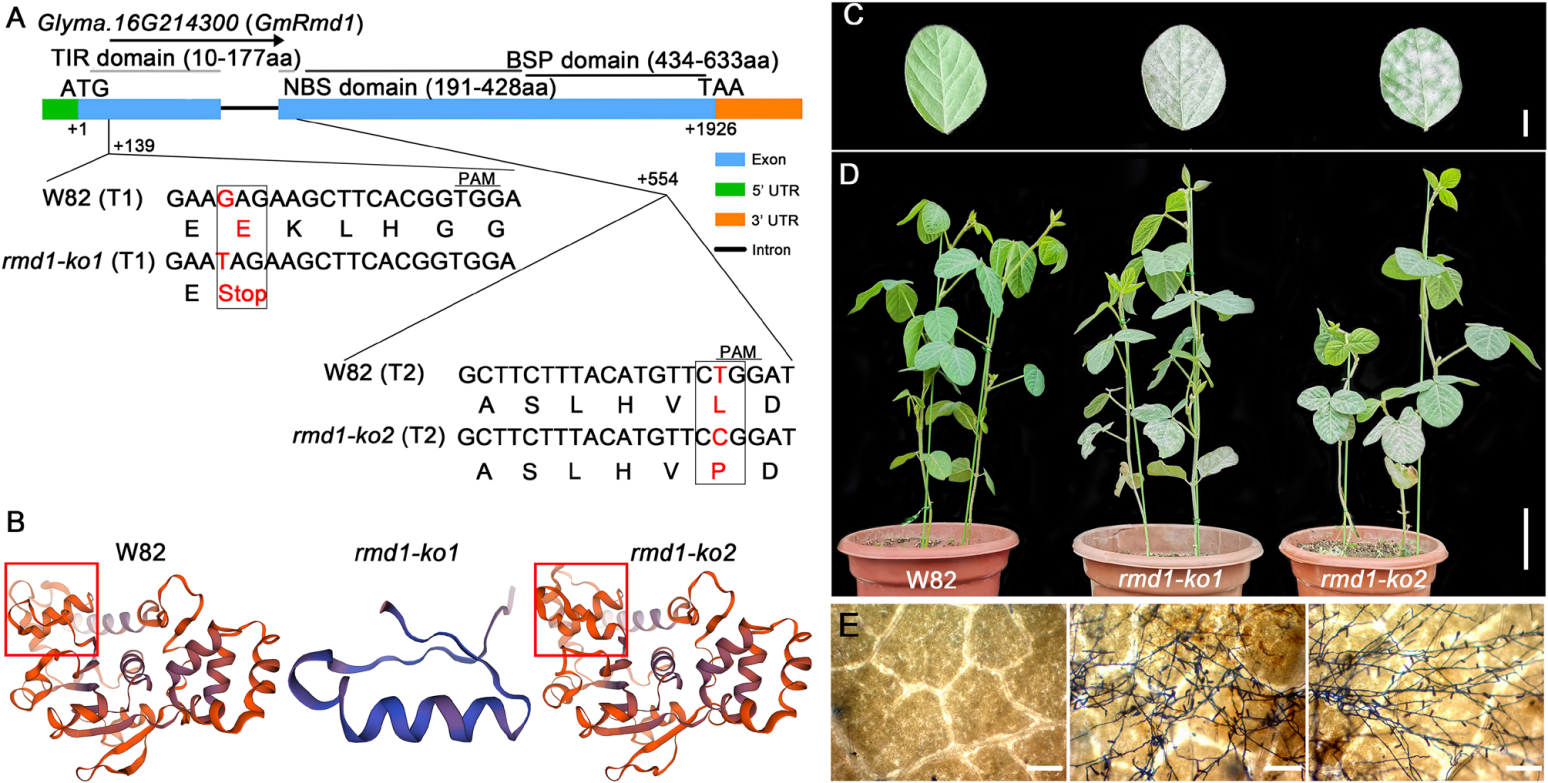
Phenotypes of the W82 and the *GmRmd1* knockout mutant. (A) Schematic diagram of the *GmRmd1* gene and its target site. (B) Comparison of the three‐dimensional structures of GmRmd1 in W82, *rmd1‐ko1* and *rmd1‐ko2*. (C, D) The leaf and plant phenotypes of W82, *rmd1‐ko1* and *rmd1‐ko2*, the scale bar = 1.5 cm in C, and the scale bar = 15 cm in D. (E) Staining of *Microsphaera diffusa* mycelium of W82, *rmd1‐ko1* and *rmd1‐ko2*, scale bar = 100 μm.

Using CRISPR/Cas9 technology to edit the *GmRmd1* in the W82 genetic background, we observed that the G‐A substitution at position 139 bp in exon 1 of *rmd1‐ko1* led to premature termination of protein translation (Figure 2A,B). Additionally, the T‐C substitution at position 554 bp in target Site 2 of *rmd1‐ko2* caused an alteration in the three‐dimensional structure of the protein (Figure 2A,B). To evaluate the function of *GmRmd1*, *M. diffusa* infection assays were conducted on W82, *rmd1‐ko1* and *rmd1‐ko2*. The results demonstrated that W82 exhibited normal growth without apparent susceptibility symptoms, whereas both *rmd1‐ko1* and *rmd1‐ko2* displayed susceptibility to *M. diffusa* , with *rmd1‐ko1* showing greater susceptibility compared to *rmd1‐ko2* (Figure 2C,D). Expression analysis revealed that the expression level of *GmRmd1* in the *rmd1‐ko1* remained consistently low both before and after infection. In contrast, the expression level of *GmRmd1* in the *rmd1‐ko2* increased following infection (Figure S5A). Subcellular localisation analysis of the GmRmd1 protein revealed that it was found mainly in the nucleus and cell membrane (Figure S5C). These findings suggest that the pathogenic phenotype observed in *rmd1‐ko1* is attributable to the knockdown of *GmRmd1*. Conversely, *rmd1‐ko2* may result from alterations in the three‐dimensional structure of GmRmd1 induced by amino acid mutations. Furthermore, cell staining analysis revealed substantial attachment of *M. diffusa* mycelium on the leaves of *rmd1‐ko1* and *rmd1‐ko2*, while minimal or no mycelium attachment was detected on W82 leaves (Figure 2E). Collectively, these results confirmed that *GmRmd1* could positively regulate PMD resistance in W82.

### 2.3 RNA‐Seq Analysis of MSRF and HX3

Leaves of MSRF and HX3 inoculated with *M. diffusa* were collected at 0, 6, 12, 24, 48 and 72 h post‐infection (hpi) for RNA extraction and reverse transcription to analyse the expression pattern of *GmRmd1* (Figure S5B). The results demonstrated that the expression of *GmRmd1* in MSRF initially increased progressively, peaked at 24 hpi and subsequently declined. In contrast, the expression level of *GmRmd1* in HX3 remained at a relatively low level throughout. This indicates that *GmRmd1* is transiently induced by *M. diffusa* .

**FIGURE 3:**
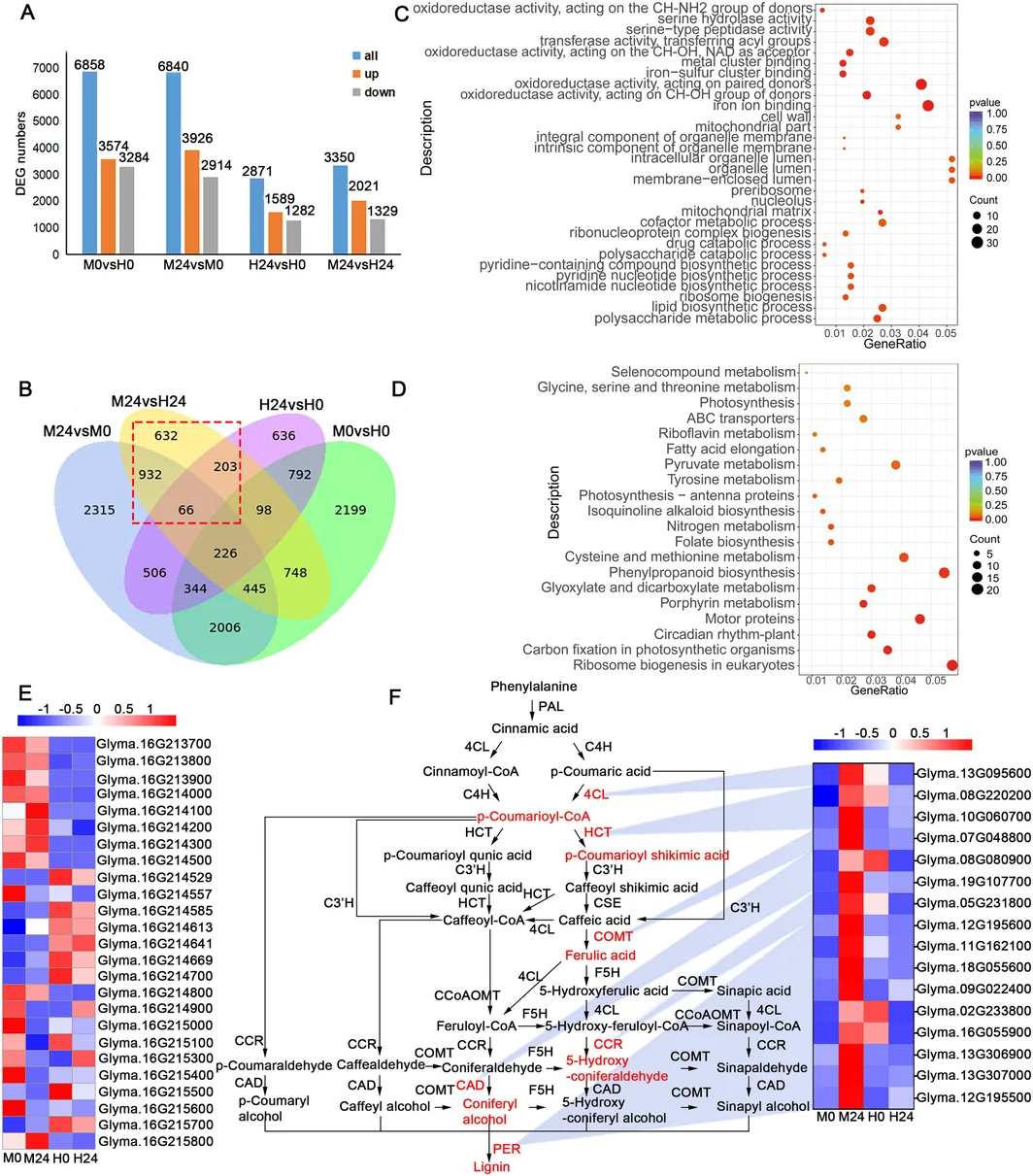
Analysis of key pathways and differentially expressed genes (DEGs) in soybean in response to *Microsphaera diffusa*. (A) The numbers of up‐regulated and down‐regulated DEGs in the four comparison groups. (B) Venn diagram of the DEGs in the four comparison groups; the red box indicates the DEGs for GO and KEGG enrichment analysis. (C) GO enrichment analysis of DEGs. (D) KEGG enrichment analysis of DEGs. (E) Expression analysis of 25 genes within the resistance gene cluster. (F) DEGs in phenylpropanoid biosynthesis pathway; Red font indicates key DEGs and their encoded enzymes.

To investigate the gene expression profiles associated with resistance to PMD in soybean, transcriptome analyses were conducted on leaves of the MSRF and the HX3 at 0 and 24 hpi with *M. diffusa* . Differentially expressed genes (DEGs) were identified in four comparison groups (M24 vs. M0, H24 vs. H0, M0 vs. H0, M24 vs. H24). The Phred quality scores Q20 in the transcriptome analysis all exceeded 90, which indicates a high level of sequencing accuracy and a low level of noise (Table S5). Principal component analysis (PCA) demonstrated that the experimental replicates within the M0, M24, H0 and H24 treatment groups exhibited good reproducibility and relatively minor intragroup variation, rendering them appropriate for subsequent differential analysis (Figure S6). A total of 12,148 genes exhibited significant changes in expression following *M. diffusa* infection (Figure 3A), with 6858 DEGs showing distinct expression patterns between the MSRF and HX3. To eliminate the potential influence of varying parental backgrounds across the four comparison groups, 1833 DEGs from the comparisons M24 versus M0, M24 versus H24 and H24 versus H0, excluding the M0 versus H0, were selected for Gene Ontology (GO) and Kyoto Encyclopedia of Genes and Genomes (KEGG) pathway enrichment analyses (Figure 3B and Table S6). The GO enrichment analysis revealed that these DEGs were predominantly associated with pathways closely linked to plant defence mechanisms, including iron–sulphur cluster binding, oxidoreductase activity and iron ion binding (Figure 3C). Similarly, KEGG enrichment analysis demonstrated that the DEGs were significantly enriched in pathways such as pyruvate metabolism, cysteine and methionine metabolism, phenylpropanoid biosynthesis, plant circadian rhythm regulation and carbon fixation, as well as other biochemical processes related to amino acid metabolism, secondary metabolite synthesis and photosynthesis (Figure 3D), all of which are known to be involved in plant defence responses.

Among the secondary metabolic pathways associated with plant disease resistance, the phenylpropanoid biosynthesis pathway holds significant importance (Dixon et al. 2002). KEGG analysis revealed that 16 DEGs were enriched in the phenylpropanoid biosynthetic pathway (Figure 3F). Among these, eight DEGs (*Glyma.10G060700*, *Glyma.07G048800*, *Glyma.19G107700*, *Glyma.12G195600*, *Glyma.11G162100*, *Glyma.18G055600*, *Glyma.13G306900* and *Glyma.13G307000*) were identified as being up‐regulated in MSRF following infection with *M. diffusa* . In contrast, their expression levels were either absent or significantly lower in HX3. The expression patterns of these genes were comparable to that of *GmRmd1*, which is known to be induced by *M. diffusa* infection. Notably, most of these DEGs are involved in lignin biosynthesis, suggesting a potentially critical role of lignin in conferring resistance against PMD.

Based on RNA‐Seq data, expression level analysis of these 25 genes within the resistance gene cluster (Liu, Fang, et al. 2024) revealed that *GmRmd1* (*Glyma.16G214300*) was up‐regulated in MSRF at 24 hpi with *M. diffusa* , whereas it showed low or no expression in HX3 (Figure 3E). The expression patterns of *Glyma.16G214100*, *Glyma.16G214200* and *Glyma.16G215800* were consistent with that of *GmRmd1*, suggesting that these genes may function synergistically in conferring resistance to PMD. In contrast, the expression levels of *Glyma.16G213700*, *Glyma.16G213800*, *Glyma.16G213900*, *Glyma.16G214000*, *Glyma.16G214500* and *Glyma.16G214800* exhibited opposite trends. These findings indicate that these genes might antagonise *GmRmd1* in regulating PMD resistance. All 25 genes were further validated by reverse transcription‐quantitative PCR (RT‐qPCR), and the results demonstrated that the expression patterns obtained from RT‐qPCR were largely consistent with those from RNA‐Seq (Figure S7), confirming the reliability and accuracy of the RNA‐Seq data.

### 2.4 *GmWRKY27* and *GmERF138* Suppress the Expression of *GmRmd1*

Through the PlantRegMap website annotation of transcription factors (https://plantregmap.gao‐lab.org) and RNA‐Seq data analysis, it was identified that the MYB transcription factor *Glyma.03G078000*, the WRKY transcription factor *Glyma.20G163200* (*GmWRKY27*) and the ERF transcription factors *Glyma.03G111700*, *Glyma.13G236600* and *Glyma.18G091600* (*GmERF138*) (Figure S8) are potentially involved in resistance to PMD. When MSRF was inoculated with *M. diffusa* for 24 h, the transcription factors *Glyma.03G078000* and *Glyma.13G236600* were significantly up‐regulated, whereas *Glyma.03G111700*, *GmWRKY27* and *GmERF138* were down‐regulated. In HX3 following 24 hpi, *Glyma.13G236600* expression was down‐regulated, while the expression levels of the other four transcription factors showed no substantial changes (Figure S8). These transcription factors exhibit expression patterns that are either similar to or opposite to that of GmRmd1 and are annotated on the PlantRegMap as defence‐related genes. Therefore, it is hypothesised that these transcription factors may participate in the regulation of *GmRmd1* expression, thereby influencing resistance to PMD.

**FIGURE 4:**
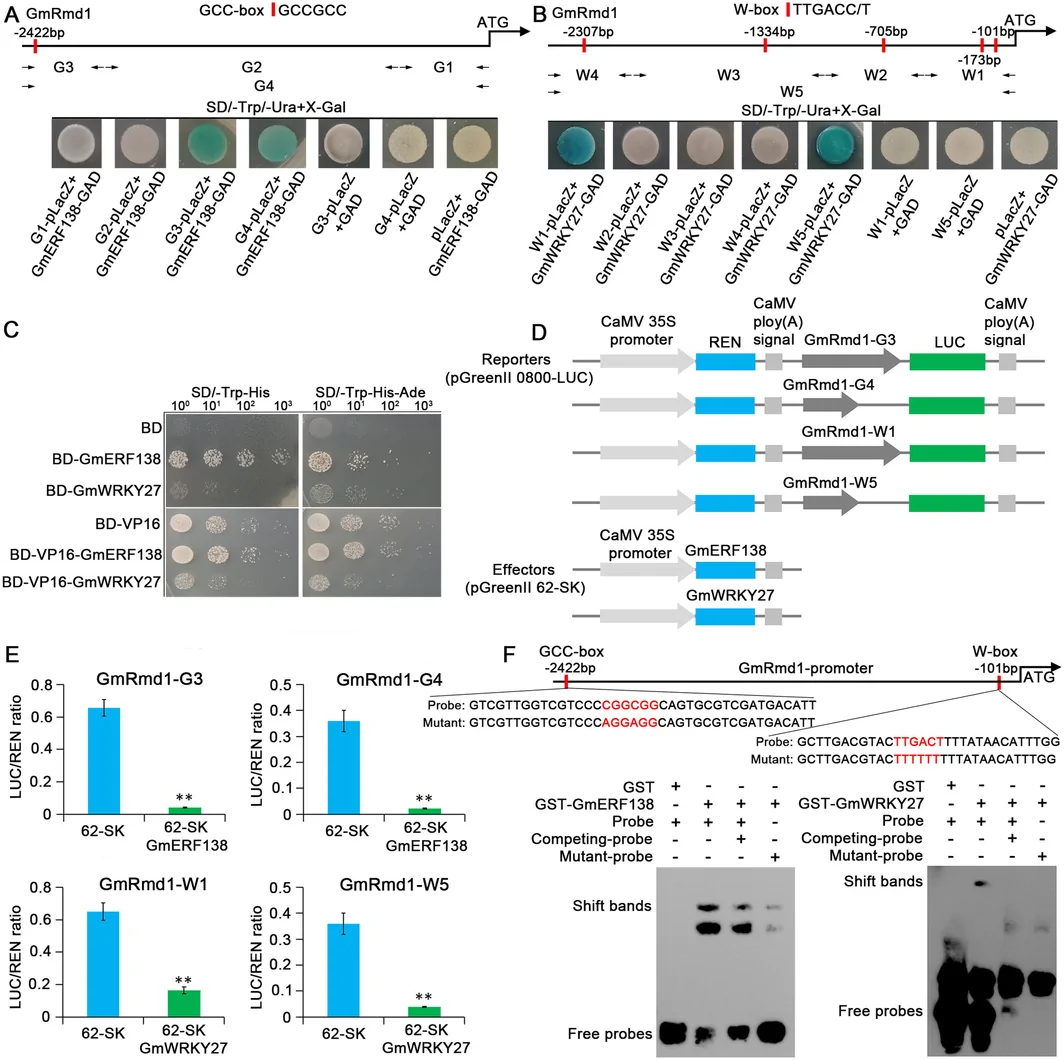
GmERF138 and *GmERF138* regulate the expression of *GmRmd1*. (A) Yeast one‐hybrid (Y1H) assay of *GmERF138* and the promoter region of *GmRmd1*, G1: −(1–500) bp of the promoter region of *GmRmd1*, G2 −(501–2000 bp), G3 −(2001–2500 bp), G5 −(1–2500 bp). (B) Y1H assay of *GmERF138* and the promoter region of *GmRmd1*, W1: −(1–500) bp of the promoter region of *GmRmd1*, W2 −(501–1000 bp), W3 −(1001–2000 bp), W4 −(2001–2500 bp) and W5 −(1–2500 bp). (C) Transcriptional activation assay of *GmERF138* and Gm*WRKY27*. (D) Diagrams illustrating the design of effector and reporter vector constructs used in the dual‐luciferase assays. (E) LUC/REN activity ratio. Six biological assays were performed. Asterisks denote significant differences: ** indicates *p* < 0.01 based on ANOVA with Tukey's correction, 62‐SK: pGreenII 62‐SK empty vector; 62‐SK *GmERF138*: *GmERF138* vector of pGreenII 62‐SK; 62‐SK *GmERF138*: *GmERF138* vector of pGreenII 62‐SK. (F) Electrophoretic mobility shift assay (EMSA) analysis of the binding interaction between GmERF138/*GmERF138* proteins and the GCC‐box/W‐box *cis*‐element probe of the *GmRmd1* promoter.

To validate the aforementioned hypothesis, the five transcription factors mentioned above were subjected to yeast one‐hybrid (Y1H) assays with *GmRmd1*. The results demonstrated that *GmERF138* bound to the GCC‐box element at position 2422 bp in the *GmRmd1* promoter region and *GmERF138* was capable of binding to the W‐box element located at either 101 bp or 173 bp within the promoter region of *GmRmd1* (Figure 4A,B), and the other three transcription factors did not exhibit binding activity to the *GmRmd1* promoter. Through yeast transcriptional activity assays, it was observed that BD‐*GmERF138* exhibited stronger growth on both double‐dropout (DDO) medium and triple‐dropout (TDO) medium compared to the BD‐empty vector (Figure 4C), indicating that *GmERF138* possesses transcriptional activation activity. In contrast, VP16‐*GmERF138* displayed weaker growth on these media relative to the BD‐VP16 empty vector (Figure 4C), suggesting that *GmERF138* exhibits transcriptional repression activity. To further elucidate whether the directly activating or repressing effects of *GmERF138* and *GmERF138* on *GmRmd1* expression, four firefly luciferase (LUC) reporter constructs were generated using the promoters GmRmd1‐G3, GmRmd1‐G4, GmRmd1‐W1 and GmRmd1‐W5, and two effector constructs were developed by co‐expressing both *GmERF138* and *GmWRKY27* (Figure 4D). The transient expression assay demonstrated that co‐expression of *GmERF138* with the GmRmd1‐G3 and GmRmd1‐G4 reporters led to a 16.0‐fold and 16.4‐fold decrease in LUC activity, respectively, compared to the control group (Figure 4E). Similarly, co‐expression of *GmERF138* with the GmRmd1‐W1 and GmRmd1‐W5 reporters resulted in a 4.0‐fold and 9.2‐fold reduction in LUC activity, respectively (Figure 4E). To further validate the aforementioned interaction, this study conducted electrophoretic mobility shift assays (EMSAs) (Figure 4F). The results indicated that the recombinant GST‐GmERF138 and GST‐*GmERF138* proteins formed detectable shifted bands when incubated with the labelled probe, while no such bands were detected with the GST‐tagged control protein (Figure 4F). Additionally, the signal intensity of the shifted bands was significantly diminished or completely eliminated upon the addition of competing or mutant probes (Figure 4F). These findings indicate that *GmERF138* and *GmERF138* act as transcriptional repressors that suppress *GmRmd1* expression by binding to the GCC‐box (5′‐GCCGCC‐3′, at the −2422 bp of the promoter) and W‐box (5′‐TTGACT‐3′, at the −101 bp of the promoter) elements in the *GmRmd1* promoter, thereby regulating PMD resistance in soybean.

### 2.5 GmRmd1 Induces Cell Death

**FIGURE 5:**
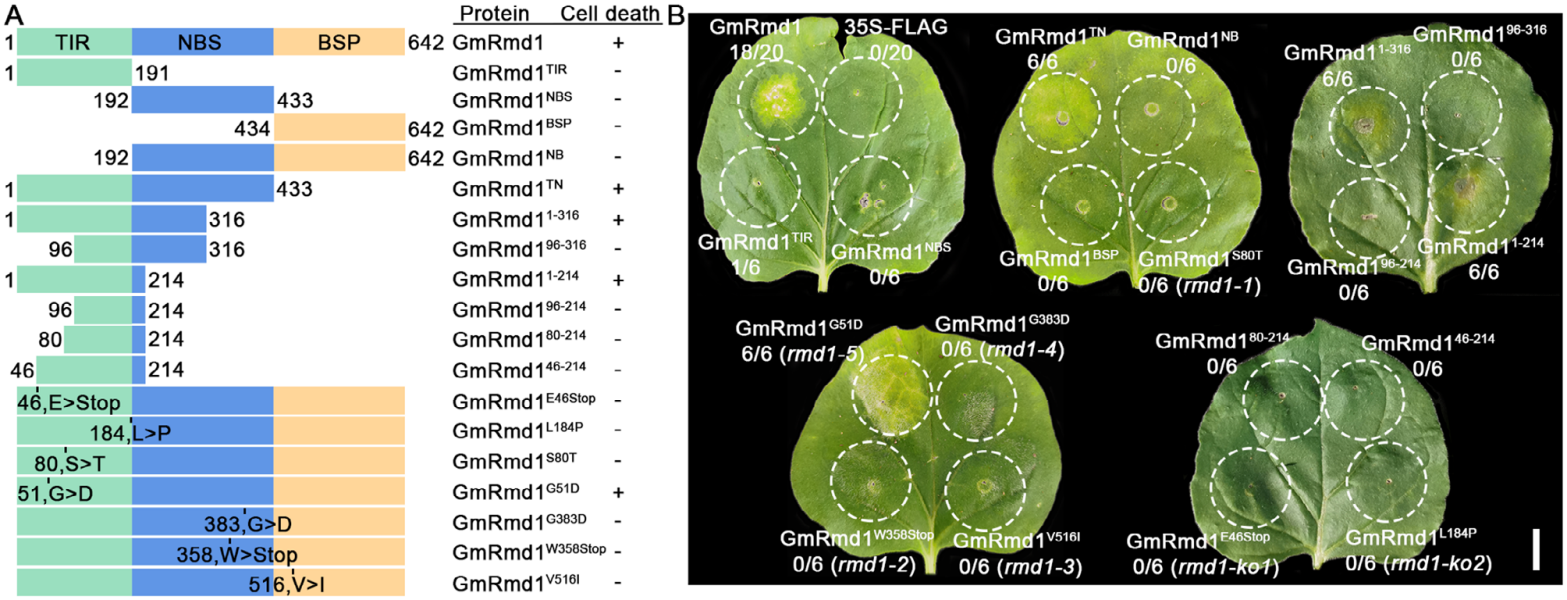
Hypersensitive response (HR) assay of GmRmd1 in *Nicotiana benthamiana*. (A) The protein structure of GmRmd1; (184, L>P) represents the 184th amino acid of GmRmd1, which is mutated from L to P, and so on. (B) The HR of *N. benthamiana*; 35S‐FLAG, empty vector control. 18/20 means that HR was induced in 18 out of 20 observations, and so on. GmRmd1^TIR^ refers to the TIR domain of GmRmd1, and so on. GmRmd1^1‐316^ represents the sequence of the GmRmd1 protein from positions 1 to 316. GmRmd1^G51D^ represents the GmRmd1 sequence derived from the W82‐ethyl methanesulfonate (EMS) mutant *rmd1‐5*; G51D means that the amino acid G at the 51th position of GmRmd1 is mutated to D, and the point mutation in *rmd1‐5* is located within the TIR domain of GmRmd1; the *rmd1‐5* confers resistance to powdery mildew disease, and so on. See Table S7 for details. Scale bar = 1 cm.

To verify the association between the hypersensitive response (HR) and PMD resistance phenotypes, MSRF, HX3, W82, *rmd1‐ko1* and *rmd1‐ko2* infected with *M. diffusa* were stained with trypan blue (Figure S10). Only a minor portion of the leaves in the resistant accessions MSRF and W82 showed cell death, while the majority of the areas in the susceptible accessions HX3, *rmd1‐ko1* and *rmd1‐ko2* presented cell death (Figure S10). This suggests that HR is correlated with disease resistance. *GmRmd1* is a nucleotide‐binding leucine‐rich repeat (NLR) gene encoding three distinct domains: TIR, NBS and BSP. Overexpression of NLR genes has been associated with the induction of HR in plant cells. To investigate whether the full‐length GmRmd1, its minimal functional unit or specific point mutations within these domains could trigger HR in *Nicotiana benthamiana*, we constructed corresponding overexpression vectors (Figure 5A) and introduced them into *Agrobacterium tumefaciens* for transient expression in *N. benthamiana* leaves. Our results demonstrated that overexpression of GmRmd1, GmRmd1^TN^, GmRmd1^1‐316^, GmRmd1^1‐214^ and the GmRmd1^G51D^ mutant induced clear HR symptoms (Figure 5B). In contrast, overexpression of GmRmd1^TIR^, GmRmd1^NBS^, GmRmd1^BSP^, GmRmd1^96‐316^, GmRmd1^96‐214^, GmRmd1^80‐214^, GmRmd1^46‐214^, GmRmd1^S80T^, GmRmd1^W358Stop^, GmRmd1^G383D^ and GmRmd1^V516I^, as well as the two knockout variants GmRmd1^E46Stop^ and GmRmd1^L184P^, did not induce the HR (Figure 5B). These results indicate that all three domains of GmRmd1 are essential for effective plant defence mechanisms and that the amino acid sequence of GmRmd1 from positions 1 to 214 might represent the minimal functional unit necessary for downstream signalling or activation of the HR cascade.

### 2.6 Self‐Interaction of GmRmd1

The GmRmd1 protein contains TIR, NBS and BSP domains (Figure S11A). To investigate whether GmRmd1 could interact with itself, a yeast two‐hybrid (Y2H) assay was conducted. The results indicated that both the control and experimental groups grew normally on the DDO medium (Figure S11B). However, AD‐GmRmd1/BD and BD‐GmRmd1/AD failed to grow on the quadruple‐dropout (QDO) medium (Figure S11B), suggesting that BD‐GmRmd1 lacked autoactivation activity. In contrast, BD‐GmRmd1/AD‐GmRmd1 exhibited normal growth on the QDO medium (Figure S11B), indicating that the full‐length GmRmd1 protein is capable of self‐interaction. Further Y2H analysis using domain‐specific constructs revealed that the TIR, NBS, BSP, TN and NB domains also supported yeast growth on the QDO medium (Figure S11B), demonstrating that both the full‐length GmRmd1 and its individual domains are able to interact in vitro.

To further verify the self‐interaction of GmRmd1 in vivo, bimolecular fluorescence complementation (BiFC) assays were conducted. Fluorescence was detectable in the nucleus for the GmRmd1‐p2YN and GmRmd1‐p2YC combination, whereas no signal was observed in the negative control (Figure S11C). Additionally, a split‐luciferase protein interaction assay was performed, revealing that LUC activity was only present when GmRmd1‐nLUC and cLUC‐GmRmd1 were co‐expressed in *N. benthamiana* leaves, as compared to the negative control (Figure S11D). Collectively, these findings indicate that GmRmd1 is capable of self‐interaction within the nucleus, and its TIR, NBS, BSP, TN and NB domains can also undergo mutual oligomerisation.

### 2.7 Interaction Between GmRmd1 and Other Proteins

**FIGURE 6:**
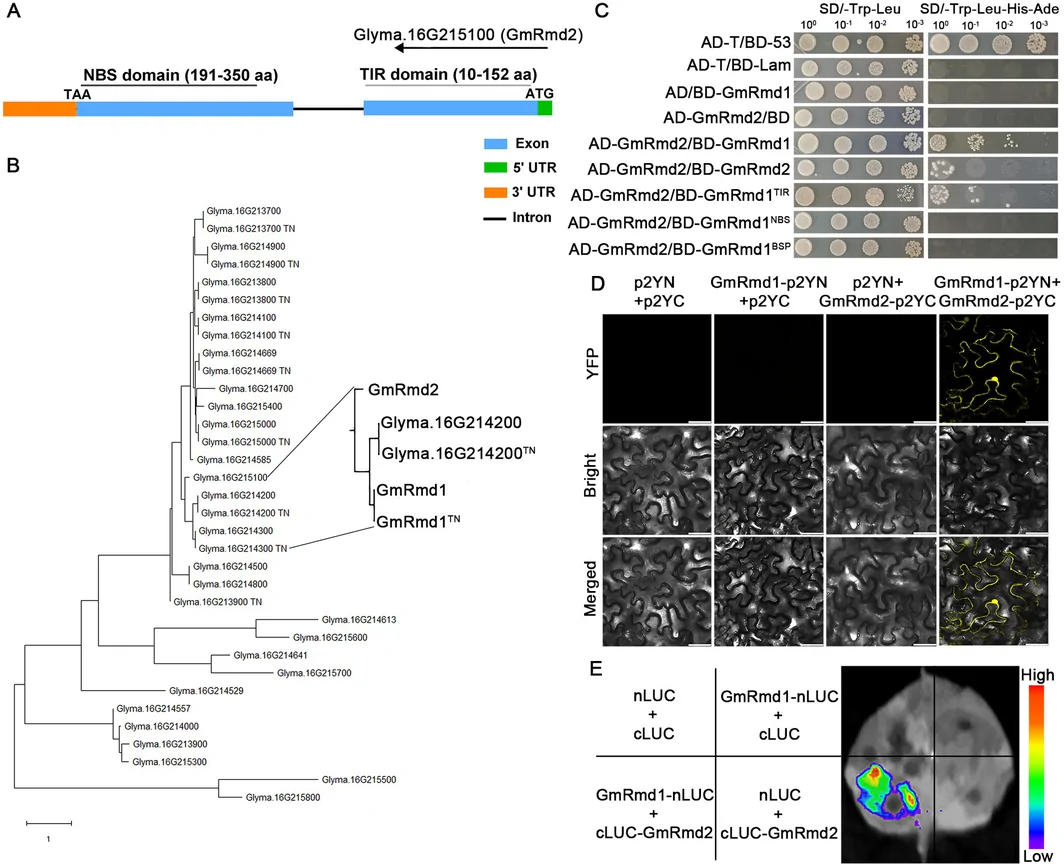
GmRmd1 forms a complex with GmRmd2. (A) The gene structure of *GmRmd2*. (B) Phylogenetic tree analysis of *GmRmd1*. (C) Yeast two‐hybrid (Y2H) assay was performed to validate the interaction between GmRmd1 and GmRmd2. (D) Bimolecular fluorescence complementation (BiFC) assay was conducted to confirm the interaction between GmRmd1 and GmRmd2. Scale bar = 20 μm. (E) Split‐luciferase (LUC) assay was carried out to verify the interaction between GmRmd1 and GmRmd2.

There are 25 genes in the chr16 terminal resistance gene cluster, of which 19 are NLR genes (Figure S12) (Liu, Fang, et al. 2024). In order to analyse the relationship between 18 NLR genes and GmRmd1, Y2H assays were performed to verify potential interactions (Figure S13). Among the 18 NLR genes, only Glyma.16G215100 (*GmRmd2*, a gene within a cluster associated with PMD resistance) and Glyma.16G214900 (*GmRmd3*, another gene within a cluster associated with PMD resistance) were found to interact with GmRmd1 (Figure 6 and Figure S14). GmRmd2 contains both TIR and NBS domains (Figure 6A). Phylogenetic tree analysis of GmRmd1 and GmRmd2 indicated that the TN domain of both proteins shares more than 80% homology (Figure 6B).

By Y2H assays, we found that AD‐GmRmd2 interacted with BD‐GmRmd2, as well as with BD‐GmRmd1 and BD‐GmRmd1^TIR^, as indicated by the growth of QDO medium (Figure 6C). To further validate these interactions, BiFC assays were conducted. The results showed that co‐expression of GmRmd1‐p2YN and GmRmd2‐p2YC led to detectable fluorescence in both the nucleus and cell membrane, whereas no fluorescence was observed in the negative control (Figure 6D). In parallel, split‐LUC assays were carried out. When GmRmd1‐nLUC and cLUC‐GmRmd2 were co‐expressed in *N. benthamiana* leaves, significant LUC activity was detected, in contrast to the negative control (Figure 6E). Taken together, these results indicate that GmRmd2 is capable of forming a homomultimer and can interact with both GmRmd1 and GmRmd1^TIR^. Both GmRmd1 and GmRmd2 are capable of forming a homomultimer.

GmRmd3 contains TIR, NBS and LRR domains (Figure S14B). Y2H assays demonstrated that AD‐GmRmd3 and BD‐GmRmd1 were able to grow on the QDO medium (Figure S14A), indicating that GmRmd3 and GmRmd1 are capable of interacting in vitro. Ethyl methanesulfonate (EMS) mutant analysis of W82 revealed that in the *rmd3‐1* mutant, the guanine base at position 174 bp within the TIR domain of GmRmd3 was substituted by thymine (Figure S14B), leading to truncated protein translation and consequently rendering *rmd3‐1* susceptible to infection by *M. diffusa* (Figure S14C,D). These findings suggest that *GmRmd3* and *GmRmd1* may share similar functional roles and both positively contribute to resistance against PMD.

Through RNA‐Seq analysis, the expression pattern of NRG1 (*Glyma.17G245600, GmRnl1*) (Figure S9), a disease resistance‐related factor containing RPW8 and NBS domains (Figure S14E), was found to be similar to that of *GmRmd1*. To investigate the potential interaction between these two proteins, a Y2H assay was performed. The results demonstrated that AD‐GmRnl1 and BD‐GmRmd1 co‐transformed yeast cells were able to grow on the QDO medium (Figure S14A), suggesting an in vitro interaction between GmRnl1 and GmRmd1. EMS mutant analysis of W82 revealed that in the *w82‐rnl1* mutant, a single nucleotide substitution from G to A at position 210 bp within the RPW8 domain of GmRnl1 caused an amino acid change (Figure S14E), leading to susceptibility to infection by *M. diffusa* (Figure S14F,G). Taken together, these findings indicate that GmRnl1 and GmRmd1 may share similar functional roles in conferring resistance against PMD.

Eight lignin synthesis‐related genes (*Glyma.10G060700*, *Glyma.07G048800*, *Glyma.19G107700*, *Glyma.12G195600*, *Glyma.11G162100, Glyma.18G055600*, *Glyma.13G306900* and *Glyma.13G307000*) were identified through RNA‐Seq analysis, and their expression patterns were consistent with that of *GmRmd1* (Figure 3F). To investigate whether these eight genes are associated with PMD resistance, we performed expression analysis. Following the *M. diffusa* infection, the expression levels of these eight genes in the knockout mutants *rmd1‐ko1* and *rmd1‐ko2* were significantly reduced compared to those in W82 (Figure S15A), suggesting that these genes are responsive to *M. diffusa* infection. To further elucidate the interaction between these eight genes and GmRmd1, an membrane system‐Y2H assay was performed (Figure S16). The results demonstrated that only Glyma.11G162100 (GmPER1, the lignin synthesis‐related gene)‐pBT3‐SUC and GmRmd1‐pPR3‐N were able to grow on the QDO medium (Figure S15B), suggesting that GmPER1 interacts with GmRmd1 outside the nucleus. By measuring the lignin content of W82 and its knockout mutants after infection with *M. diffusa* for 7 days, we found that the lignin content in *rmd1‐ko1* and *rmd1‐ko2* was significantly lower than that in W82 (Figure S15C). These results suggest that *GmRmd1* and *GmPER1* may mediate resistance to PMD by regulating lignin biosynthesis.

### 2.8 The Geographical Distribution of *GmRmd1* in 315 Soybean Accessions

**FIGURE 7:**
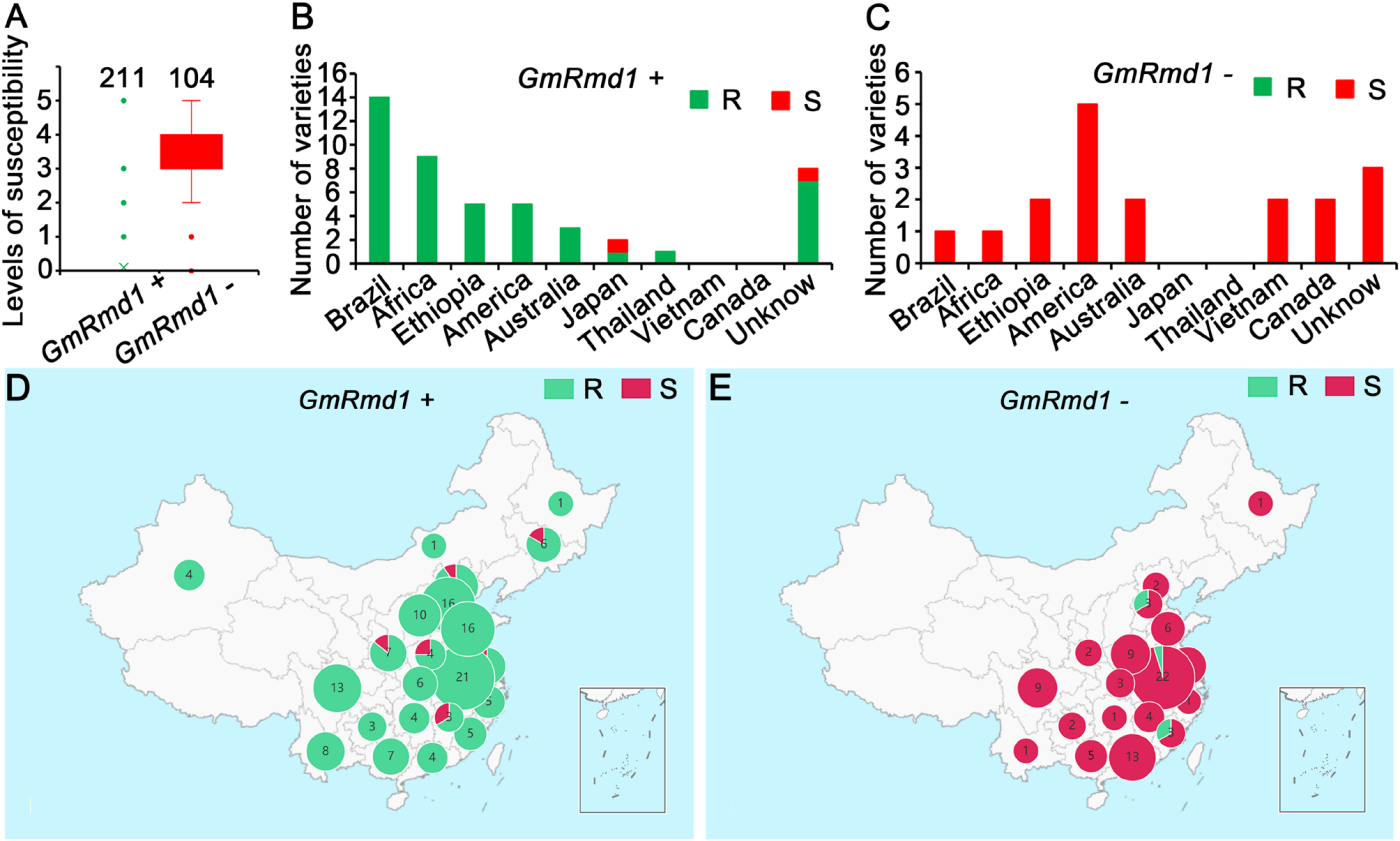
Geographical distribution of *GmRmd1* in 315 soybean accessions. (A) The box plot of accessions that are resistant and susceptible to powdery mildew disease with complete and missing *GmRmd1; GmRmd1*+, the coding region of *GmRmd1* is intact; *GmRmd1*−, *GmRmd1* is incomplete. (B, C) Distribution of resistant and susceptible accessions to powdery mildew disease in different countries under *GmRmd1* complete (B) and missing (C) conditions. (D, E) Distribution of resistant and susceptible accessions to PMD in different regions of China with complete (D) and missing (E) *GmRmd1*.

We evaluated the relationship between the integrity of the *GmRmd1* coding region and PMD resistance in 315 soybean accessions (Table S8) (Liu, Fang, et al. 2024), and found that 211 accessions had a complete *GmRmd1* coding region, of which 96.2% (203 of total 211) were related to PMD resistance (Figure 7A). Only 3.8% (8 of total 211) of accessions have susceptible phenotypes and also have an intact *GmRmd1* locus. They are from Japan (1), one accession of unknown origin, Jilin (1), Beijing (1), Jiangsu (1), Henan (1), Jiangxi (1) and Shaanxi (1) (Figure 7B,D). By comparison, we found 104 accessions with *GmRmd1* deletions, of which 97.2% (101 of total 104) are susceptible and only 2.8% (3 of total 104) are resistant (Figure 7C,E). The resistant accessions with disrupted *GmRmd1* loci are from Anhui (1), Fujian (1) and Hebei (1). The most sensitive accessions have L3 or L4 degrees of susceptibility (Figure 7A and Table S8). In conclusion, the number of *GmRmd1* complete accessions gradually decreased from northern to southern China, and a similar trend was observed for resistant accessions. This suggests that the complete *GmRmd1* plays a critical role in conferring resistance to PMD. Therefore, the selection and utilisation of soybean accession with a complete *GmRmd1* coding region could serve as an effective strategy to mitigate the risks associated with PMD in breeding programmes.

### 2.9 Variation Types and Geographical Distribution of *GmRmd1* in Worldwide Soybean Accessions

To investigate the geographic distribution of *GmRmd1* on a global scale, whole‐genome resequencing data PRJCA002030 of 2898 accessions was downloaded from the national genomics data centre (https://ngdc.cncb.ac.cn/) (Figure S17). We found that there are rich PMD resistance resources around the world and that *GmRmd1* genotypes are generally clustered into four groups, with groups 1 and 4 having intact *GmRmd1* loci (Figure S17A). These two groups are mostly distributed in Europe and northeastern China (Figure S17B,C). In China, 73.4% of accessions have an intact *GmRmd1* locus (1615 of total 2200). Interestingly, the percentages of accessions with intact *GmRmd1* loci decrease gradually from north to south and west to east (Figure S17C). Northeastern China, including Shanxi with 94.6% (157 of total 166), Hebei with 87.6% (85 of total 97), Jilin with 82.1% (147 of total 179) and Liaoning with 81.1% (107 of total 132), have high percentages of a complete *GmRmd1* (Figure S17C). By contrast, accessions from southeastern China such as those from Jiangsu 52.2% (59 of total 113), Jiangxi 36.4% (4 of total 11), Guangdong 57.1% (12 of total 21), and Sichuan 55.0% (22 of total 40) have a complete *GmRmd1* (Figure S17C). Additionally, South Korea, Russia and Canada have relatively low percentages of a complete *GmRmd1* at 53.8% (35 of total 65), 48.1% (25 of total 52) and 23.8% (5 of total 21), respectively (Figure S17B). Soybeans are most susceptible to PMD at reproductive stages, and cool weather late in the growing season is most conducive to disease development (Jun et al. 2012). Therefore, these results suggest that *GmRmd1* may be subject to selection. Intact *GmRmd1* loci were observed more frequently in accessions from cool climate regions such as northern China, where soybeans are planted during the spring with a higher risk of PMD infection.

## 3 Discussion

The majority of resistance (R) proteins in plants are NLR proteins. These proteins exhibit conserved structures, typically comprising a nucleotide‐binding site (NBS), a leucine‐rich repeat (LRR) domain and an N‐terminal domain (Duxbury et al. 2016; Jacob et al. 2013). Based on the N‐terminal domain, plant NLR can be classified into three categories: TNL, CNL and RNL. The TIR/CC domain of NLRs is responsible for transmitting immune signals, while the NBS domain plays a critical role in ATP/ADP binding and exhibits AAA‐ATPase‐like enzymatic activity, functioning as a molecular switch to connect immune sensing with signalling. Additionally, the LRR domain recognises effectors secreted by pathogenic bacteria and regulates NLR activity through intramolecular and intermolecular interactions (Sun et al. 2020; Saur et al. 2021). In addition, non‐regular domains are increasingly recognised in plant NLR proteins and have been demonstrated to play a crucial role in pathogen recognition or immune signalling (Guo et al. 2018; Marchal et al. 2018; Wang et al. 2020). GmRmd1 is an atypical TNL protein containing three domains: TIR, NBS and BSP. Transient expression assays in *N. benthamiana* indicated that the TN domain of GmRmd1 plays a predominant role in triggering the HR. However, single‐point mutations in any of the three domains can abolish HR induction in *N. benthamiana*, suggesting that all three domains are functionally essential in defence against PMD.

This study revealed that the GmRmd1 and GmRmd2 proteins form homo‐oligomers via self‐interaction. Studies have demonstrated that the self‐oligomerisation of NLR proteins constitutes a critical step in their functional activation. Atypical TIR domain‐containing proteins can form oligomers upon induction by substrates such as NAD^+^ or ATP, thereby activating NAD^+^ hydrolase activity and initiating immune responses (Song et al. 2024). ZAR1 maintains its oligomeric state via ADP binding. When ATP binding is induced by effector factors, the protein undergoes conformational rearrangements and assembles into a pentameric resistosome, thereby initiating an immune response (Sun et al. 2020). In addition, NLR proteins can regulate plant immune homeostasis through self‐oligomerisation. In tomato, SlNRC2 exists in an inactive state as dimers and tetramers, with its C‐terminal LRR domain interacting with inositol phosphates IP6 and IP5 to stabilise this inactive conformation. Upon pathogen infection, effector proteins secreted by the pathogen induce the dissociation of these oligomers, thereby activating the immune response (Ma, An, et al. 2024). A similar mechanism has been identified in the wheat NLR protein Pm6Sl, where the CC‐BED domain suppresses autoimmunity through self‐oligomerisation and is relieved from inhibition only upon pathogen recognition (Ma, Tian, et al. 2024). These findings elucidate the dynamic regulatory mechanism underlying NLR self‐interaction and offer novel insights into the structural basis of immune activation.

Genes within paired or larger NLR clusters function synergistically to trigger immunity and are required to form dimers or multimers to mediate downstream immune responses (Wersch and Li 2019). GmRmd1 lacks the LRR domain responsible for recognising the *M. diffusa* , with its LRR domain replaced by a BSP domain, suggesting that GmRmd1 relies on the cooperative activity of other proteins to mediate its disease resistance function. In this study, in addition to its self‐interaction, GmRmd1 also forms interactions with both GmRmd2 and GmRmd3 proteins. Their genes are located within a disease resistance gene cluster at the distal end of chr16. During infection by *M. diffusa* , these proteins interact with each other to activate immune responses. NLR genes are commonly organised in genomic clusters, and functional synergy among their members plays a crucial role in conferring effective disease resistance and generating diversity in resistance mechanisms. In the wheat PMD resistance gene cluster, the head‐to‐head oriented NLR genes *RXL* and *Pm5e* form a complex. The atypical coiled‐coil (CC) domain of Pm5e precisely modulates the intensity and specificity of the immune response through competitive inhibition of RXL overactivation (Guo et al. 2025). The *Arabidopsis* NLR proteins RPS4 and RRS1 are essential for interacting to recognise the bacterial effector AvrRps4 in *Pseudomonas syringae* and PopP2 in *Xanthomonas aquatilis*, thereby triggering plant immunity (Sun et al. 2020). Similarly, pairs of NLR genes in rice, such as *Pit1*/*Pit2* (Li et al. 2024), *Pib*/*PibH8* (Wang et al. 2024) and *RGA4*/*RGA5* (Césari et al. 2014), function synergistically to confer resistance against *Magnaporthe oryzae*. In soybean rust resistance research, the NLR proteins Rpp6907‐4 and Rpp6907‐7 exhibit antagonistic roles, balancing the trade‐off between disease resistance and plant growth (Hao et al. 2024).

The gene interactions within the disease resistance gene cluster are mainly located at the distal end of chr16. However, RNA‐Seq and molecular assay identified a potential interaction between GmRnl1 and GmRmd1 on chr17. GmRnl1 belongs to the RNL gene family and contains both the RPW8 domain (a resistance‐related domain originally identified in *Arabidopsis* PMD proteins) and the NBS domain. It typically functions as an NRG1‐type resistance‐assisting factor contributing to disease resistance (Saile et al. 2020). The interaction between GmRnl1 and GmRmd1 may align with the TNL‐ADR1/NRG1 cooperative model described in dicotyledonous plants, wherein the sensor NLR protein (GmRmd1) recruits the helper NLR protein (GmRnl1) to form a complex that amplifies immune signals and mediates defence responses (Xiao et al. 2025; Huang et al. 2025). In *Arabidopsis*, the formation of the EDS1‐SAG101‐NRG1 ternary complex represents a critical step in TNL‐mediated signalling pathways, suggesting that the NLR‐RNL interaction identified in this study might reflect a similar functional cooperation mechanism in legumes (Xiao et al. 2025). In the context of multigene cooperative regulation of disease resistance, tomato NRC2 (SlNRC2) forms high‐order oligomers with other members of the NRC family (NRC3 and NRC4) to support the functionality of various sensor NLRs and mediate calcium signalling (Liu, Yang, et al. 2024). This two‐gene synergistic mechanism not only enhances immune robustness but also reduces the risk of resistance loss caused by mutations in a single gene. Furthermore, the co‐evolution of genes within the resistance cluster may contribute to maintaining functional equilibrium through epigenetic regulatory mechanisms.

Transcription factors play a crucial role in the regulation of plant immune signalling pathways. In this study, two transcription factors associated with plant defence responses, *GmERF138* and *GmWRKY27*, were identified through RNA‐Seq analysis. Both *GmERF138* and *GmERF138* are capable of specifically binding to the GCC‐box and W‐box *cis*‐regulatory elements, respectively, within the promoter region of *GmRmd1*, thereby suppressing expression of *GmRmd1*. Previous studies have demonstrated that certain transcription factors can positively regulate NLR gene expression during immune activation. For instance, in the case of the TNL protein N, which confers resistance to tobacco mosaic virus, the transcription factors *NRZ1* and *NRM1* directly bind to the promoter of protein N and enhance its expression, subsequently triggering downstream immune responses (Zhang et al. 2024). However, research investigating transcription factors that directly interact with NLR promoters to modulate immune responses remains limited. This study reveals that *GmERF138* and *GmERF138* can bind to the *GmRmd1* promoter and regulate its expression, which contributes significantly to addressing this research gap.

**FIGURE 8:**
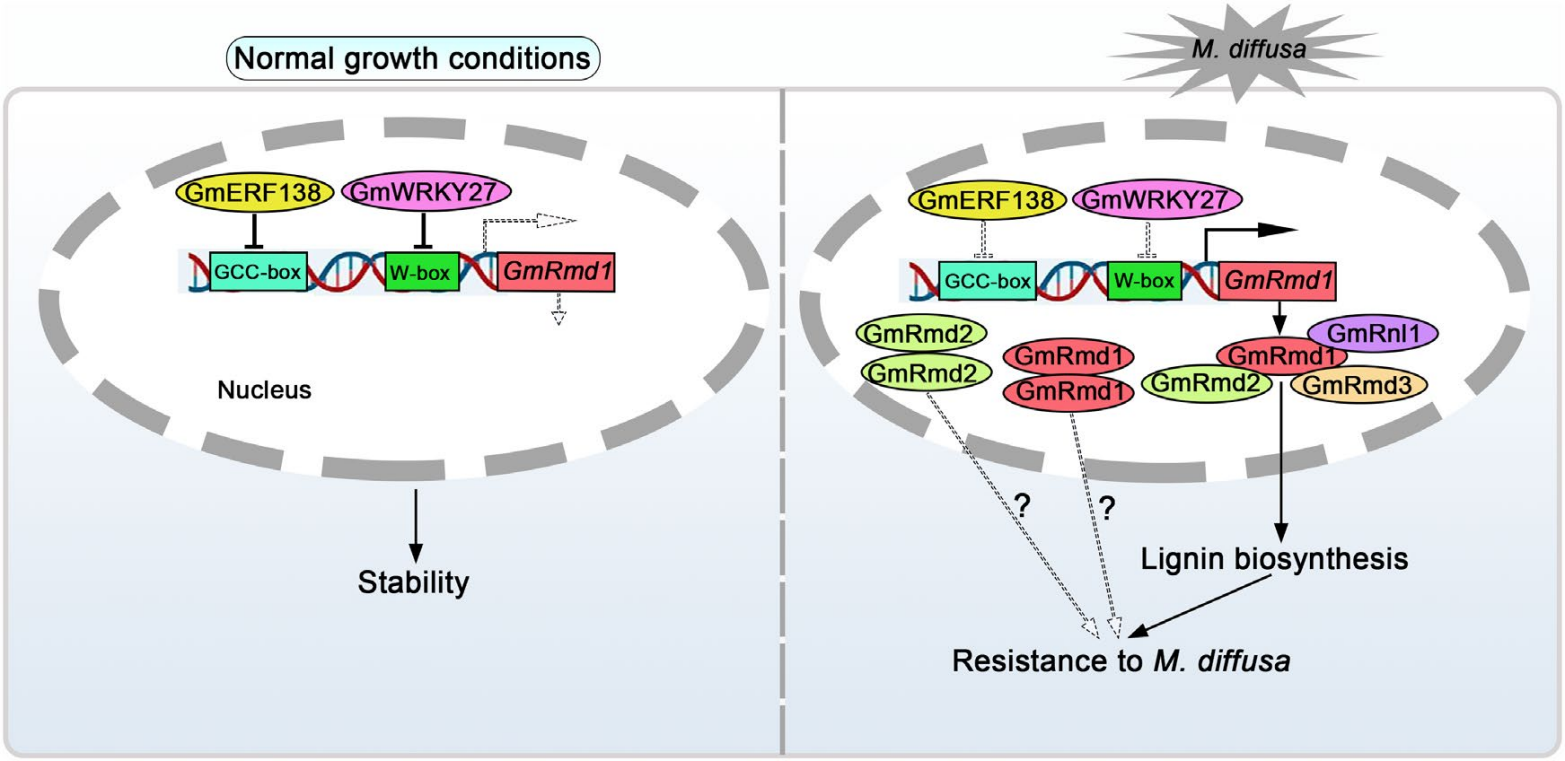
Molecular mechanism of *GmRmd1* regulating powdery mildew disease of soybean.

Based on the findings of this study, a model diagram illustrating the resistance mechanism of soybean PMD is proposed (Figure 8). In the absence of infection by *M. diffusa* , *GmERF138* and *GmERF138* bind specifically to the GCC‐box and W‐box *cis*‐regulatory elements within the promoter region of *GmRmd1*, thereby suppressing its expression. Upon infection with *M. diffusa* , the expression of *GmRmd1* is relieved by *GmERF138* and *GmERF138*. Both GmRmd1 and GmRmd2 form homodimers through self‐interaction, thereby conferring disease resistance. GmRmd1 forms a complex with resistance‐related proteins such as GmRmd2, GmRmd3 and GmRnl1, and the complex can regulate the expression of genes associated with lignin biosynthesis, thereby promoting lignin production and enhancing resistance against *M. diffusa* . This study has achieved notable progress in elucidating the regulatory mechanism of PMD mediated by the *GmERF138*/GmERF138‐GmRmd1/GmRmd2/GmRmd3/GmRnl1 module. These findings provide novel insights into the molecular basis of *GmRmd1*‐mediated PMD resistance in soybean, as well as valuable genetic resources for future molecular breeding efforts aimed at improving resistance to PMD.

## 4 Experimental Procedures

### 4.1 Plant Materials and Evaluation of PMD Resistance (for Fine Mapping)

MSRF, HX3 and F_2_–F_6_ generations of the MSRF × HX3 population (abbreviated as MH population) were maintained in our laboratory. MSRF is resistant to PMD, and HX3 is a highly susceptible PMD cultivar. The hybrid population and its parental lines were planted in the soybean experimental field at the Zengcheng Experimental Base of South China Agricultural University in December 2021, December 2022 and December 2023. The plants were exposed to natural infection by *M. diffusa* , and the disease status of the leaves was systematically recorded. Each line was planted in three rows with a spacing of 15 cm between plants and 40 cm between rows. Single‐seed sowing was performed with three replicates for each treatment.

### 4.2 DNA Extraction and Gene Mapping

DNA was extracted from young leaves at V2 stage (second trifoliate stage) using the sodium dodecyl sulphate (SDS) method (Perry 2004). Gene sequences were downloaded from the Phytozome database (https://www.phytozome.org). Primers were designed using SnapGene software (https://www.snapgene.com/). The primer length was approximately 20 bp, with an annealing temperature of 56°C and GC content ranging from 40% to 50%. Primers used for gene mapping are shown in Table S2. All primers used in this study were synthesised by Sangon Biotech (Shanghai) Co. Ltd. The above primers were used to perform PCRs using DNA from the population of location. The PCR conditions were (1) 94°C for 2 min; (2) 33 cycles of 94°C for 30 s, 50°C for 30 s and 72°C 20 s and (3) 72°C for 5 min. PCR products were detected using 6% nondenaturing polyacrylamide gel electrophoresis.

### 4.3 Functional Verification

This study used the target‐design module of the gene editing website CRISPR‐GE (http://skl.scau.edu.cn/home/) to analyse *GmRmd1*, and designed corresponding primers based on the recommended target sites (Target 1: 5′‐ATACAGGGCTCTCTGTGAGAAGG‐3′ and Target 2: 5′‐CGAGCTTCTTTACATGTTCTGG‐3′). Subsequent genetic transformation was carried out by Wuhan Aidijing Biotech Co. Ltd., ultimately resulting in wild‐type Williams 82 (W82, resistant to PMD) and *GmRmd1* knockout mutants (*rmd1‐ko1* and *rmd1‐ko2*). The EMS mutant of W82 was kindly donated by Nanjing Agricultural University (http://isoybean.org/).

W82, the *GmRmd1* knockout mutant, the EMS mutant of W82, MSRF and HX3 were cultivated in the greenhouse at South China Agricultural University in December 2023 and December 2024. The mutant lines underwent two generations of self‐crossing to generate homozygous lines corresponding to candidate genes. Each material was planted in two pots, with five plants per pot. When the first trifoliate leaf was fully expanded, spores were collected from diseased leaves in the soybean experimental field and used to prepare a spore suspension for artificial inoculation. Following infection, the mycelium was stained for further analysis (Xian et al. 2022).

### 4.4 Analysis of Gene Expression

The treatment of soybean leaves and the analysis of gene expression levels were conducted following the methods of previous studies (Liu, Fang, et al. 2024). Soybean plants at the V1 stage were inoculated with a spore suspension of *M. diffusa* containing 1 × 10^5^ CFU/mL and grown in a growth chamber maintained at 23°C, 75% relative humidity and a 16 h light/8 h dark photoperiod. The experiment was conducted in three biological replicates. Leaves were collected at 0, 24 and 72 hpi and stored at −80°C for subsequent RNA‐Seq. Total RNA was extracted using the HiPure Plant RNA Mini Kit (Magen), and 1 mg of total RNA was reverse‐transcribed into first‐strand cDNA employing the Evo MMLV RT Kit with gDNA Clean (Accurate Biology). The expression levels at various time points subsequent to infection were detected via fluorescence‐based quantitative PCR (qPCR) Sangon Biotech (Shanghai) Co. Ltd. Quantitative data were analysed in accordance with the Bio‐Rad Fluorescence qPCR Application Guide, and the relative expression levels of the target gene were presented as 2^−Δ*C*t^ or 2^−ΔΔ*C*t^ values. The specific primers for real‐time fluorescence qPCR are shown in Table S9.

### 4.5 Transcriptome Analysis by RNA‐Seq

MSRF and HX3 leaves stored at −80°C (inoculated with *M. diffusa* for 0 and 24 hpi) were used for RNA‐Seq. The sample codes are as follows: M24 for samples infected with resistant *M. diffusa* , and corresponding control samples labelled as M0; H24 for samples infected with susceptible materials' *M. diffusa* , and corresponding control samples labelled as H0. The collected samples were promptly frozen in liquid nitrogen and stored at −80°C for subsequent RNA extraction, to be used in library sequencing. RNA‐seq library construction was prepared by Novogene (Tianjin, China) Co. Ltd. and an Illumina NovaSeq 6000 with the paired‐end 1 × 150 bp model was used for sequencing. The clean reads were mapped to the reference *G. max* Wm82.a4.v1 genome (https://www.soybase.org/) using Hisat2 (v. 2.0.5). StringTie (v. 1.3.3b) was used for transcript assembly (Pertea et al. 2015), and differential gene expression was analysed employing DEGseq. We used |log_2_fold change| ≥ 2 and *p*_adj_ < 0.05 as threshold conditions (Han et al. 2025; Han et al. 2019). DEGs were screened in four comparison groups (M24 vs. M0, H24 vs. H0, M0 vs. H0, M24 vs. H24).

### 4.6 Subcellular Localisation

The 1305‐GFP vector backbone was double‐digested using the restriction enzymes BamHI and KpnI. Primers containing homologous arms (Table S9) were designed, and the *GmRmd1* coding sequence was amplified via PCR using cDNA from W82 as the template. The resulting PCR product was then ligated into the digested 1305‐GFP vector to generate the 1305‐GmRmd1‐GFP fusion expression vector. This recombinant vector was subsequently introduced into *Agrobacterium tumefaciens* GV3101 competent cells (Shanghai Weidi Biotechnology Co. Ltd.). Following transformation, *Agrobacterium*‐mediated infiltration was performed on *N. benthamiana* leaves. After 2 days of incubation under dark conditions at 21°C, subcellular localisation was analysed using a laser scanning confocal microscope. To facilitate cellular compartment identification, pUC19/mChe (expressing mCherry) was used as a nuclear and cytoplasmic marker, while pUC19/mChe‐PM served as a plasma membrane marker (Han et al. 2022).

### 4.7 Yeast Hybridisation Assays

The Y2H, membrane system yeast two‐hybrid (m‐Y2H), Y1H and yeast transcriptional activation assays were performed using EcoRI and BamHI as the restriction enzyme sites. The plasmids pGADT7 (AD) and pGBKT7 (BD), pPR3‐N and pBT3‐SUC, pLacZi2μ and pJG4‐5, as well as pGBKT7 and pGBKT7‐VP16 were subjected to double digestion. Homologous arm primers were designed to amplify the coding sequence of the target gene (Table S9). The resulting PCR products were cloned into the respective vectors to generate fusion expression constructs. These recombinant vectors were co‐transformed into competent *Saccharomyces cerevisiae* strains AH109 (for Y2H), NMY51 (for m‐Y2H), EGY48 (for Y1H) and Y2HGold (for yeast transcriptional activation assay) and subsequently cultured on double dropout media (DDO) SD−Leu−Trp (for Y2H and m‐Y2H) or SD−Trp−Ura (for Y1H) and on single‐dropout medium (SDO) SD−Trp (for yeast transcriptional activation assay) at 28°C for approximately 2 days. Positive monoclonal colonies were selected and cultured in the corresponding liquid medium at 28°C with shaking at 200 rpm for 14 h. A 10 μL aliquot of the bacterial suspension was serially diluted 10‐, 100‐ and 1000‐fold, and then spotted on the SD−His−Leu−Trp and SD−Ade−His−Leu−Trp (Y2H and m‐Y2H), SD−Trp−Ura and SD/−Trp/−Ura/Gal/Raf/X‐Gal (Y1H) and SD−His−Trp and SD−Ade−His−Trp (yeast transcriptional activation assay) media. The inoculated media were incubated at 28°C for 3–4 days, and yeast growth was monitored. Results were interpreted according to the manufacturer's instructions (Beijing Coolaber Technology Co. Ltd.).

### 4.8 EMSA

The open reading frames of *GmERF138* and *GmWRKY27* were cloned into the pGEX‐4T‐1 vector to construct expression vectors for GST‐GmERF138 and GST‐*GmERF138*. Primers are shown in Table S9. Subsequently, the recombinant plasmids were transformed into *Escherichia coli* BL21 cells and induced with 0.2 mM isopropyl β‐d‐thiogalactoside (IPTG) to express GST‐GmERF138 and GST‐*GmERF138* proteins. The GST‐tagged proteins were purified using the corresponding GST purification kit (Solarbio). The EMSA probes were labelled with 5′‐biotin (Figure 4F). The EMSA experiments were performed using a chemiluminescent EMSA kit (ThermoFisher), in accordance with the manufacturer's instructions.

### 4.9 Trypan Blue Staining Assay

Naturally infected leaves were collected from different accessions. 70% ethanol was added and the leaves incubated in a 60°C water bath for 60 min. After the incubation, the ethanol was discarded and the leaves rinsed twice with distilled water, with each rinse lasting 30 s. A 0.4% trypan blue staining solution was added and the leaves incubated in a 60°C water bath for 10–15 min. After staining, the staining solution was discarded and a decolourising solution composed of ethanol and glycerol in a 1:1 ratio was added. The leaves were decolourised at room temperature for 1 h until the background was clean and the blue‐stained cells were clearly observable. The decolourised leaves were placed on a microscope slide, glycerol added and photographs taken under a microscope for record‐keeping.

### 4.10 HR Assay in *N. benthamiana*

The pTF101‐FLAG vector was used as the backbone for conducting the HR assay. Initially, the pTF101‐FAG vector was digested using restriction enzymes PacI and SpeI. Subsequently, the coding sequence of the target gene was amplified via PCR using homologous arm primers (Table S9). The resulting PCR product was then cloned into the pTF101‐FLAG vector to generate a fusion expression vector containing the target gene. This fusion vector was introduced into *A. tumefaciens* GV3101 through transformation. *Agrobacterium* suspensions carrying different plasmids were then combined in equal volumes and infiltrated into *N. benthamiana* leaves. Following infiltration, photos were taken and recorded after culturing in the dark for 2 days.

### 4.11 BiFC Assay

The p2YN and p2YC vectors were double‐digested using PacI and SpeI as restriction sites. Subsequently, homologous arm primers were designed to amplify the coding sequence of the target gene (Table S9). The resulting PCR products were separately inserted into the p2YN and p2YC vectors to generate fusion expression vectors carrying the target gene. These fusion vectors were then transformed into *A. tumefaciens* GV3101. Equal volumes of the two *Agrobacterium* suspensions, each harbouring a different plasmid, were mixed and infiltrated into *N. benthamiana* leaves. The infiltrated leaves were incubated in the dark at 21°C for 2 days. Finally, fluorescence signals at the infiltration sites were examined and captured using laser confocal microscopy.

### 4.12 Split LUC Complementation Assay

The pCAMBIA1300‐cLUC and pCAMBIA1300‐nLUC vectors were digested with KpnI and SalI as restriction sites, and then homologous arm primers were designed to amplify the coding sequence of the target gene (Table S9). These amplified coding sequences were then ligated into the respective vectors via homologous recombination. The resulting fusion vectors were introduced into *A. tumefaciens* GV3101 through transformation. Negative controls and experimental groups were established for comparison. Two or three *Agrobacterium* suspensions carrying different plasmids were mixed in equal volumes and infiltrated into *N. benthamiana* leaves. The inoculated leaves were incubated in the dark at 21°C for approximately 2 days. Following transformation, the leaves were uniformly applied with LUC substrate (8 mg/50 mL, Promega) and kept in darkness for 5 min to allow substrate absorption. The leaves were then placed into a plant live‐imaging system (LB985 Night SHADOW) and allowed to equilibrate for 2 min prior to fluorescence imaging. The luminescence signals were captured and recorded (Wang et al. 2021).

### 4.13 Dual‐Luciferase Assay

The pGreenII 0800LUC and pGreenII 62‐SK vectors were double‐digested using XhoI/BamHI and XbaI/EcoRI, respectively. Homologous arm primers were subsequently designed to amplify the coding sequence of the target gene (Table S9). The coding sequence of the target gene was ligated to the corresponding vector using homologous recombination enzymes, and the constructed fusion vectors were transformed into *A. tumefaciens* GV3101. Both negative controls and experimental groups were prepared and co‐infiltrated into *N. benthamiana* leaves. Following infiltration, the plants were incubated in darkness at 21°C for approximately 2 days. LUC activity was quantified by measuring firefly LUC and Renilla luciferase (REN) signals using a dual‐luciferase reporter assay kit (Vazyme) on a microplate reader (Sense 425‐301, Hidex). The experiment was performed with four biological replicates.

### 4.14 Lignin Quantification Assay

The lignin content was determined using the acetyl bromide method, as previously described (Chang et al. 2008). Approximately 70–80 mg of soybean leaf tissue infected by the *M. diffusa* for 7 days was weighed and dried at 80°C. Subsequently, approximately 3 mg of cell wall extract was weighed, and the lignin content was quantified using a lignin content detection kit (Sangon Biotech (Shanghai) Co. Ltd.) in accordance with the manufacturer's instructions. The prepared solution was transferred to a 96‐well microplate, and the absorbance was measured at 280 nm using a microplate reader. The extinction coefficient of lignin used for quantification was 23.35 mL/mg/cm^2^.

### 4.15 Bioinformatics Analysis

For promoter analysis, the 2500 bp sequence upstream of the start codon of the candidate gene was retrieved from the Phytozome database (https://phytozome‐next.jgi.doe.gov). *Cis*‐element analysis was performed using the PlantCARE tool (https://bioinformatics.psb.ugent.be/webtools/plantcare/html/). The results obtained from PlantCARE were then imported into TBtools (https://github.com/CJ‐Chen/TBtools‐II/releases) for visualisation.

Amino acid sequences were aligned using the NCBI website (https://www.ncbi.nlm.nih.gov/) to download candidate genes. Sequence alignment was performed in MEGA (https://www.megasoftware.net/). To compare the results, DeneDoc (https://nrbsc.org/gfx/genedoc/) was used for the analysis of the genetic structure of conserved regions.

The target protein sequence was uploaded to the Swiss‐Model platform (https://swissmodel.expasy.org) for evolutionary modelling. A model was established from the initial data, and the three‐dimensional structure model with the highest confidence level was selected based on comparative analysis. The structural features of the protein were visualised using PyMOL (http://pymol.org). For phylogenetic analysis, the *G. max* Wm82.a4.v1 genome (https://phytozome‐next.jgi.doe.gov) was used to retrieve homologous protein sequences of GmRmd1, and 50 sequences with high homology were selected from the database. Subsequently, a phylogenetic tree was constructed using the neighbour‐joining (NJ) method in MEGA‐X (https://www.megasoftware.net).

## Author Contributions

**Guoqiang Liu:** conceptualization, methodology, data curation, formal analysis, validation, investigation, software, writing – original draft, writing – review and editing. **Mengshi Liu:** data curation, formal analysis, conceptualization. **Jiacan Jiang:** software, methodology, validation, investigation. **Jiajian Zhang:** investigation, validation. **Yunxin He:** conceptualization. **Lu Li:** data curation, formal analysis, investigation. **Chen Ling:** methodology, validation, investigation. **Xiaomin Xu:** investigation, validation, methodology. **Xuehui Zhang:** investigation, validation. **Bing Hu:** conceptualization, writing – review and editing, writing – original draft. **Tingting Xu:** methodology, validation. **Cunyi Yang:** conceptualization, data curation, supervision, funding acquisition, visualization, project administration, resources, writing – original draft, writing – review and editing.

## Funding

This work was supported by the open competition programme of top ten critical priorities of Agricultural Science and Technology Innovation for the 14th Five‐Year Plan of Guangdong Province (Grant 2022SDZG05), the National Natural Science Foundation of China (Grants 32172061 and 32000403), Yuelushan Laboratory Talent Program (Grant 2025RC2022) and Guangdong Laboratory of Lingnan Modern Agriculture.

## Conflicts of Interest

The authors declare no conflicts of interest.

## Supporting information


**Figure S1:** Phenotypes of the MSRF and HX3 after being infected by *Microsphaera diffusa* at the seedling and the mature stage.

**Figure S2:** Genotype of markers Satt431 and InDel516514 in F_2_ offspring of the MH population.

**Figure S3:** IGV visualisation of the fine‐mapping interval in MSRF and HX3.

**Figure S4:** Protein sequence alignment of GmRmd1 in W82 (Williams 82), MSRF, B13 and HX3.

**Figure S5:** Relative expression levels of *GmRmd1*.

**Figure S6:** Principal component analysis of M0, M24, H0 and H24.

**Figure S7:** Expression analysis of 25 genes within the resistance gene cluster by reverse transcription‐quantitative PCR.

**Figure S8:** Expression analysis of transcription factors.

**Figure S9:** Expression analysis of disease resistance cofactor‐related genes.

**Figure S10:** Trypan blue staining of leaves infected with *Microsphaera diffusa* in different resistance accessions.

**Figure S11:** The self‐interaction of GmRmd1.

**Figure S12:** The domains of 25 candidate genes within the gene cluster.

**Figure S13:** Yeats two‐hybrid analysis of 18 NLR genes with GmRmd1.

**Figure S14:** The interaction of GmRmd1 with GmRmd3 and GmRnl1.

**Figure S15:** Lignin mediated resistance to powdery mildew disease.

**Figure S16:** Yeast two‐hybrid analysis of lignin biosynthesis‐related genes with GmRmd1.

**Figure S17:** The types of genetic variation of *GmRmd1* in 2898 soybean accessions and their geographical distribution.

**Table S1:** Segregation patterns of MH population resistance to powdery mildew disease in the F_2_, *F*_2:3_.

**Table S2:** The DNA markers and primers used for mapping.

**Table S3:** Phenotype and genotype information of F_2_ population of MH.

**Table S4:** Fine mapping of the powdery mildew disease resistance gene in the F_2_, F_4_ and F_5_ populations of MH.

**Table S5:** The raw data quality and alignment rates of the transcriptome analysis.

**Table S6:** Detailed description of 1833 differentially expressed genes.

**Table S7:** Analysis of the mutation sites of GmRmd1 in the ethyl methansulfonate (EMS) and knockout mutants of W82.

**Table S8:** The information of 315 accessions in soybean.

**Table S9:** Primers and purposes used in this study.

## Data Availability

The raw sequences of transcriptome were deposited in the CNCB with the accession number PRJCA043827 (https://www.cncb.ac.cn/search?dbId=&q=PRJCA043827). The data that support the findings of this study are available in the Supporting Information of this article.
