# Supplementary material for: Transcriptional and Functional Insights Into the All‐Stage Resistance Gene GmRmd1‐Mediated Regulation of Resistance to Powdery Mildew in Soybean
Source: Mol Plant Pathol. 2026 Aug 28;27(9):e70330. doi: 10.1111/mpp.70330 (PMC13525195; doi:10.1111/mpp.70330)
Supplement: Supplementary file 1 [file MPP-27-e70330-s002.docx]

**Figure S10 Trypan blue staining of leaves infected with *M. diffusa* in different resistance accessions**

Note: R: Resistant, S: Susceptible, the scale bar=1.5 cm.
