# Supplementary material for: Transcriptional and Functional Insights Into the All‐Stage Resistance Gene GmRmd1‐Mediated Regulation of Resistance to Powdery Mildew in Soybean
Source: Mol Plant Pathol. 2026 Aug 28;27(9):e70330. doi: 10.1111/mpp.70330 (PMC13525195; doi:10.1111/mpp.70330)
Supplement: Supplementary file 2 [file MPP-27-e70330-s003.docx]

**Figure S2 Genotype of markers Satt431 and InDel516514 in F_2_ offspring of the MH population**

Note: **(A)** Genotype of markers Satt431 in F_2_ offspring of the MH population. M stands for MSRF, and H stands for HX3. The size maker is 200 bp. The MSRF PCR product is 185 bp and that of HX3 is 244 bp. Numbers 1, 6, 7, etc., represent F_2_ offspring of the MH population. **(B)** Genotype of markers InDel516514 in F_2_ offspring of the MH population. The size maker is 180 bp. The MSRF PCR product is 198 bp, and the HX3 PCR product is 247 bp. Numbers 1, 6, 7, etc., represent F_2_ offspring of the MH population.
