# Supplementary material for: Transcriptional and Functional Insights Into the All‐Stage Resistance Gene GmRmd1‐Mediated Regulation of Resistance to Powdery Mildew in Soybean
Source: Mol Plant Pathol. 2026 Aug 28;27(9):e70330. doi: 10.1111/mpp.70330 (PMC13525195; doi:10.1111/mpp.70330)
Supplement: Supplementary file 3 [file MPP-27-e70330-s004.docx]

**Figure S15 Lignin mediated resistance to PMD**

Note: **(A)** Analysis of the expression levels of genes related to lignin synthesis; the relative expression level was set with the expression level of W82 at 7 day post-infection with *M. diffusa* as the control; *GmActin* was used as the internal reference, and the error bars indicate the standard deviation of three biological replicates, n=3; * and ** means significant difference determined using the student’s *t*-test at *P*<0.05 and *P*<0.01, respectively. **(B)** m-Y2H assay of GmRmd1 and GmPER1. **(C)** Determination of lignin content in W82 and its knockout mutants; DW, dry weight (oven drying). Values are means ±SD (n=3). ***P* <0.01 (Student’s *t*-test).
