# Supplementary material for: Transcriptional and Functional Insights Into the All‐Stage Resistance Gene GmRmd1‐Mediated Regulation of Resistance to Powdery Mildew in Soybean
Source: Mol Plant Pathol. 2026 Aug 28;27(9):e70330. doi: 10.1111/mpp.70330 (PMC13525195; doi:10.1111/mpp.70330)
Supplement: Supplementary file 4 [file MPP-27-e70330-s005.docx]

**Figure S14 The interaction of GmRmd1 with GmRmd3 and GmRnl1**

Note: **(A)** Y2H assay was conducted to verify the interaction relationship between GmRmd1 and GmRmd3 as well as GmRnl1. **(B)** The gene structure of *GmRmd3* and its mutation types; G > T, Homozygous, G > Stop: The base G is mutated to base T, a homozygous mutant, and the amino acid G is mutated, and translation is terminated prematurely. **(C, D)** The PMD phenotypes of the leaves and plants of W82 and the EMS mutant *rmd3-1* of W82, the scale bar=1.5 cm of C, and the scale bar=10 cm of D. **(E)** The gene structure of *GmRnl1* and its mutation types. **(F, G)** The PMD phenotypes of the leaves and plants of W82 and the EMS mutant *rnl1-1* of W82. the scale bar=1.5 cm of F, and the scale bar=10 cm of G.
