# Supplementary material for: Transcriptional and Functional Insights Into the All‐Stage Resistance Gene GmRmd1‐Mediated Regulation of Resistance to Powdery Mildew in Soybean
Source: Mol Plant Pathol. 2026 Aug 28;27(9):e70330. doi: 10.1111/mpp.70330 (PMC13525195; doi:10.1111/mpp.70330)
Supplement: Supplementary file 6 [file MPP-27-e70330-s008.docx]

**Figure S11 The self-interaction of GmRmd1**

Note: **(A)** The protein structure of GmRmd1. **(B)** Y2H to validate the interaction relationships between GmRmd1 itself and its domains. TN: TIR and NBS domains of GmRmd1; NB: NBS and BSP domains of GmRmd1. **(C)** BiFC was used to verify the interaction of GmRmd1 itself; Scale bar=20 μm. **(D)** Split LUC was employed to verify the self-interaction of GmRmd1.
