# Supplementary material for: Transcriptional and Functional Insights Into the All‐Stage Resistance Gene GmRmd1‐Mediated Regulation of Resistance to Powdery Mildew in Soybean
Source: Mol Plant Pathol. 2026 Aug 28;27(9):e70330. doi: 10.1111/mpp.70330 (PMC13525195; doi:10.1111/mpp.70330)
Supplement: Supplementary file 7 [file MPP-27-e70330-s009.docx]

**Figure S7 Expression analysis of 25 genes within the resistance gene cluster by RT-qPCR**

Note: *GmActin* was used as the internal reference, and the error bars indicate the standard deviation of three biological replicates, n =3.
