# Supplementary material for: Transcriptional and Functional Insights Into the All‐Stage Resistance Gene GmRmd1‐Mediated Regulation of Resistance to Powdery Mildew in Soybean
Source: Mol Plant Pathol. 2026 Aug 28;27(9):e70330. doi: 10.1111/mpp.70330 (PMC13525195; doi:10.1111/mpp.70330)
Supplement: Supplementary file 9 [file MPP-27-e70330-s012.docx]

**Figure S5 Relative expression levels of *GmRmd1***

Note: **(A)** Expression levels of *GmRmd1* in W82 and the *rmd1-ko* mutant at different stages after being infected by *M. diffusa*. CK: Control group when not infected by *M. diffusa*, SS: Seedling stage, MS: Mature stage. **(B)** Expression levels of *GmRmd1* in MSRF and HX3 accessions at different time periods after being infected by *M. diffusa*; *GmActin* was used as the internal reference, and the error bars indicate the standard deviation of three biological replicates, n =3; ** means significant difference determined using student’s *t*-test at *P*<0.01. **(C)** Subcellular localization of the GmRmd1 protein. Marker for the cell nucleus and cell membrane, Scale bar=10 μm.
