# Supplementary material for: Transcriptional and Functional Insights Into the All‐Stage Resistance Gene GmRmd1‐Mediated Regulation of Resistance to Powdery Mildew in Soybean
Source: Mol Plant Pathol. 2026 Aug 28;27(9):e70330. doi: 10.1111/mpp.70330 (PMC13525195; doi:10.1111/mpp.70330)
Supplement: Supplementary file 16 [file MPP-27-e70330-s023.docx]

**Figure S4 Protein sequence alignment of *GmRmd1* in W82 (**[**Williams 82**](https://baike.baidu.com/item/Williams%2082/57866535)**), MSRF, B13, and HX3**
