# Supplementary material for: Transcriptional and Functional Insights Into the All‐Stage Resistance Gene GmRmd1‐Mediated Regulation of Resistance to Powdery Mildew in Soybean
Source: Mol Plant Pathol. 2026 Aug 28;27(9):e70330. doi: 10.1111/mpp.70330 (PMC13525195; doi:10.1111/mpp.70330)
Supplement: Supplementary file 17 [file MPP-27-e70330-s024.docx]

**Figure S17 The types of genetic variation of *GmRmd1* in 2898 soybean accessions and their geographical distribution**

Note: **(A)** Variation types of *GmRmd1* in 2898 accessions. Genotype code of *GmRmd1* in all 2898 accessions were clustered into four groups, which are heterozygote (pink color), alternative homozygote (red color), missing (grey color) and reference homozygote (green color). **(B)** Distribution of 2898 accessions with complete and missing *GmRmd1* in the world. **(C)** Distribution of 2200 accessions with complete and missing *GmRmd1* in China. Numbers in pie charts represent the total number of accessions locate in that country **(B)** or province of China **(C)**. Green and red color represent accessions with complete *GmRmd1* (*GmRmd1* +) and missing *GmRmd1* (*GmRmd1* -), respectively.
