# Supplementary material for: Transcriptional and Functional Insights Into the All‐Stage Resistance Gene GmRmd1‐Mediated Regulation of Resistance to Powdery Mildew in Soybean
Source: Mol Plant Pathol. 2026 Aug 28;27(9):e70330. doi: 10.1111/mpp.70330 (PMC13525195; doi:10.1111/mpp.70330)
Supplement: Supplementary file 21 [file MPP-27-e70330-s016.docx]

**Figure S13 Y2H analysis of 18 NLR genes with GmRmd1**
